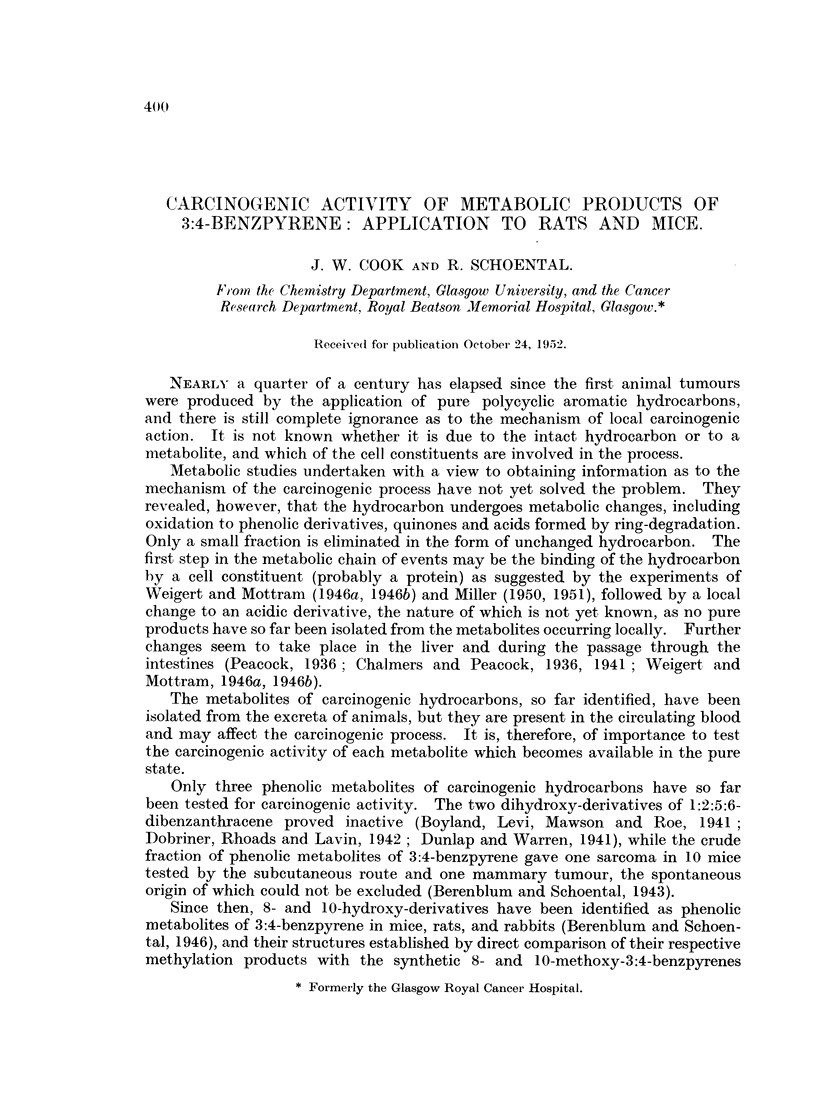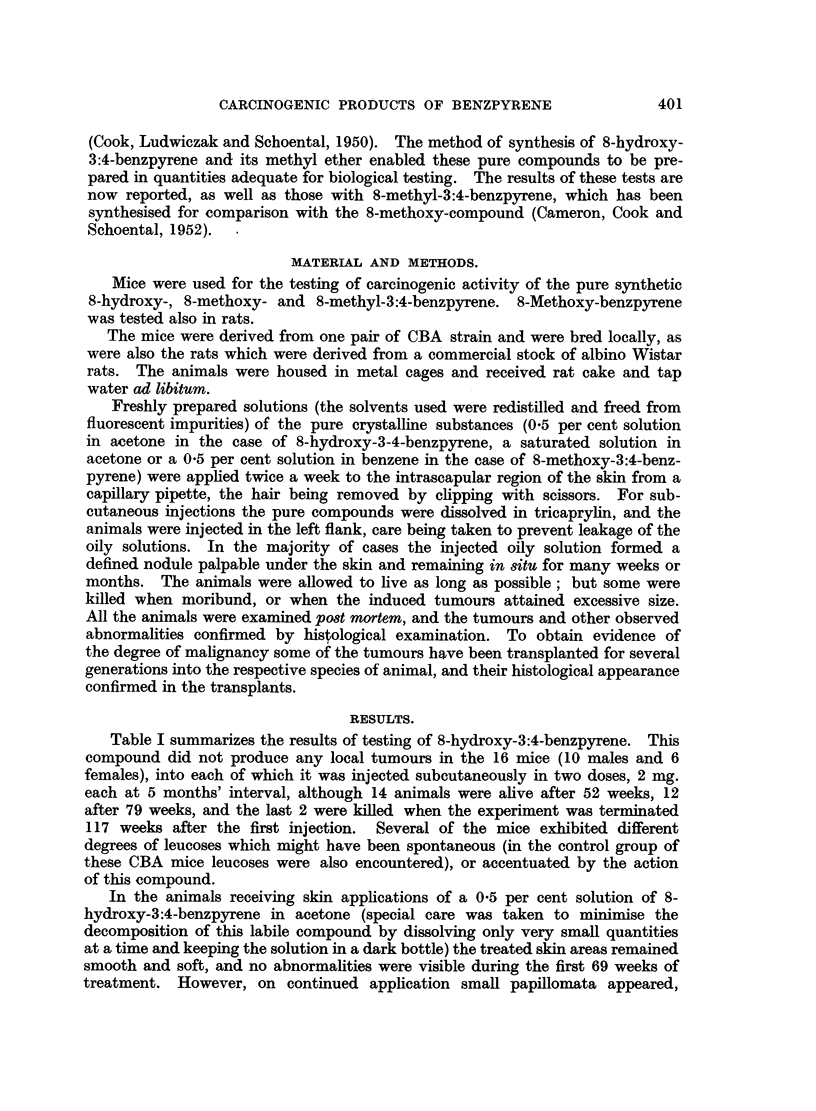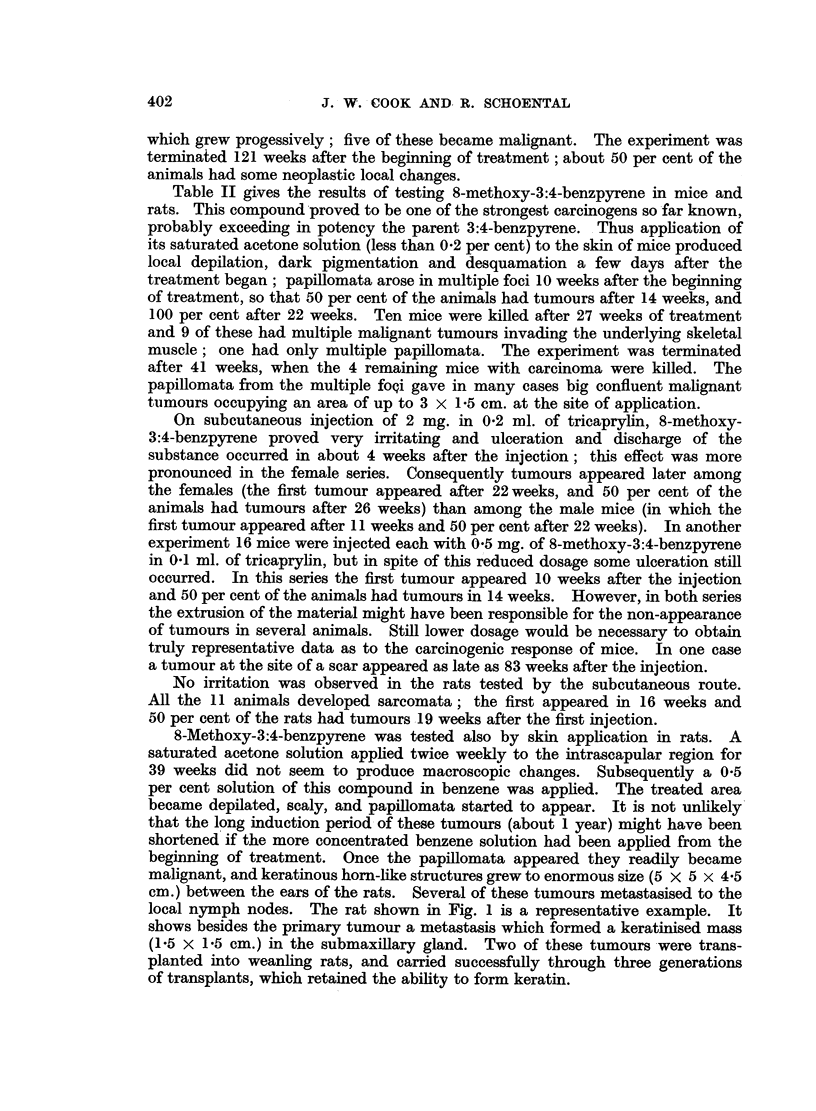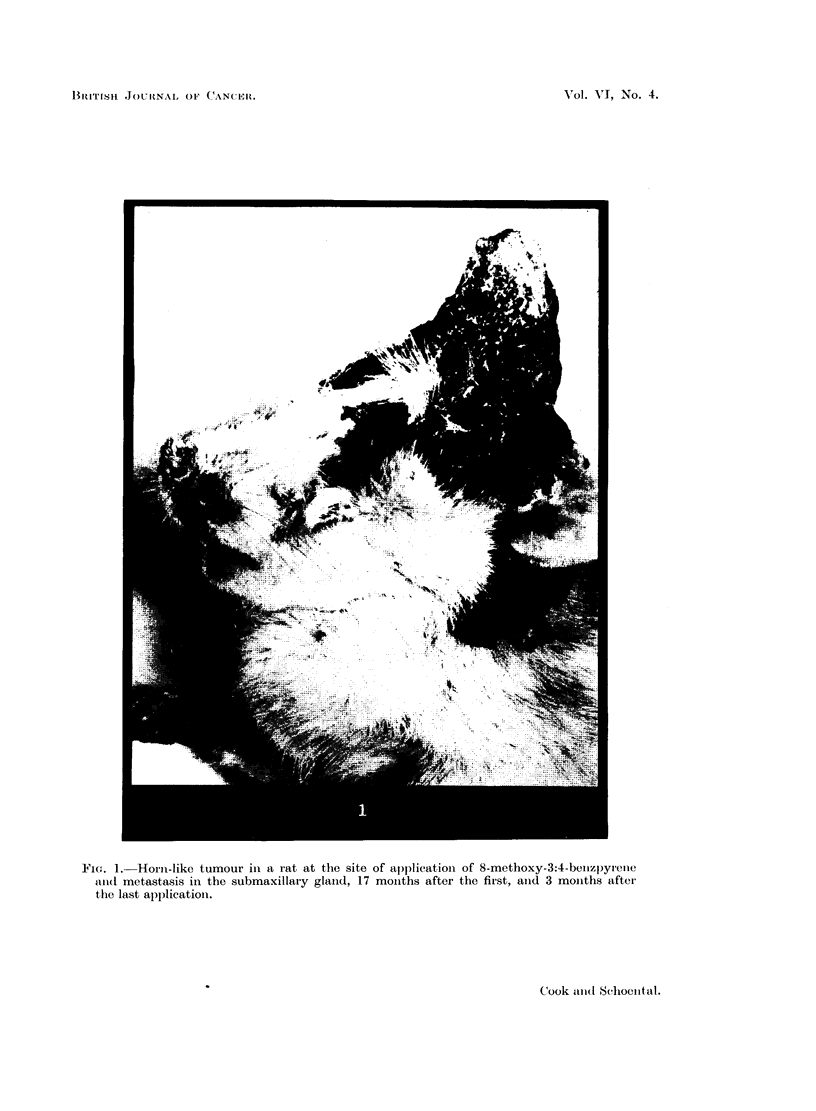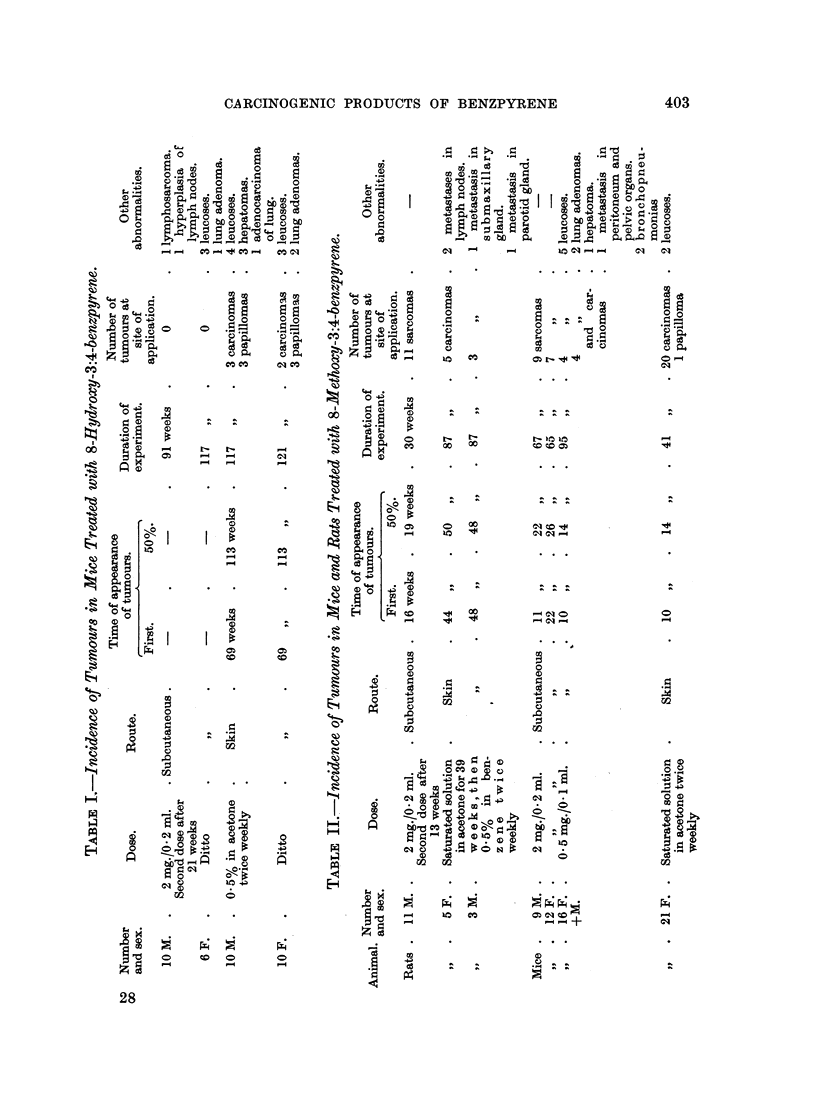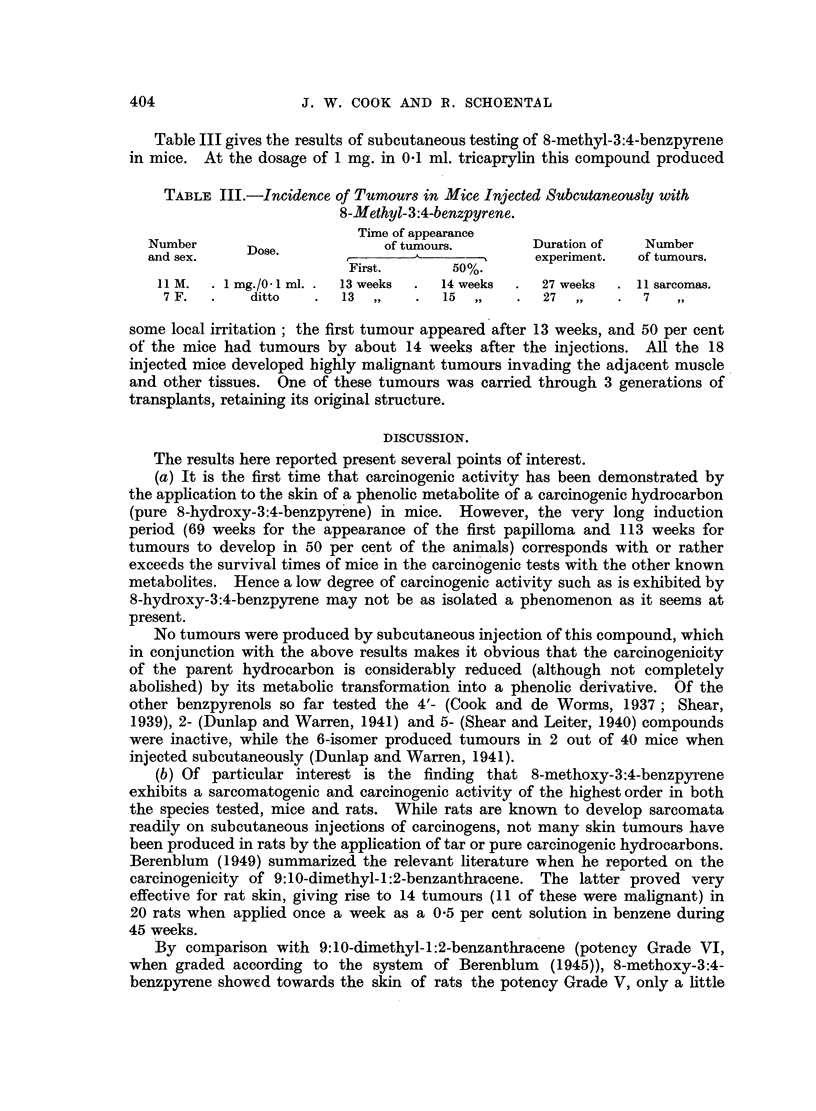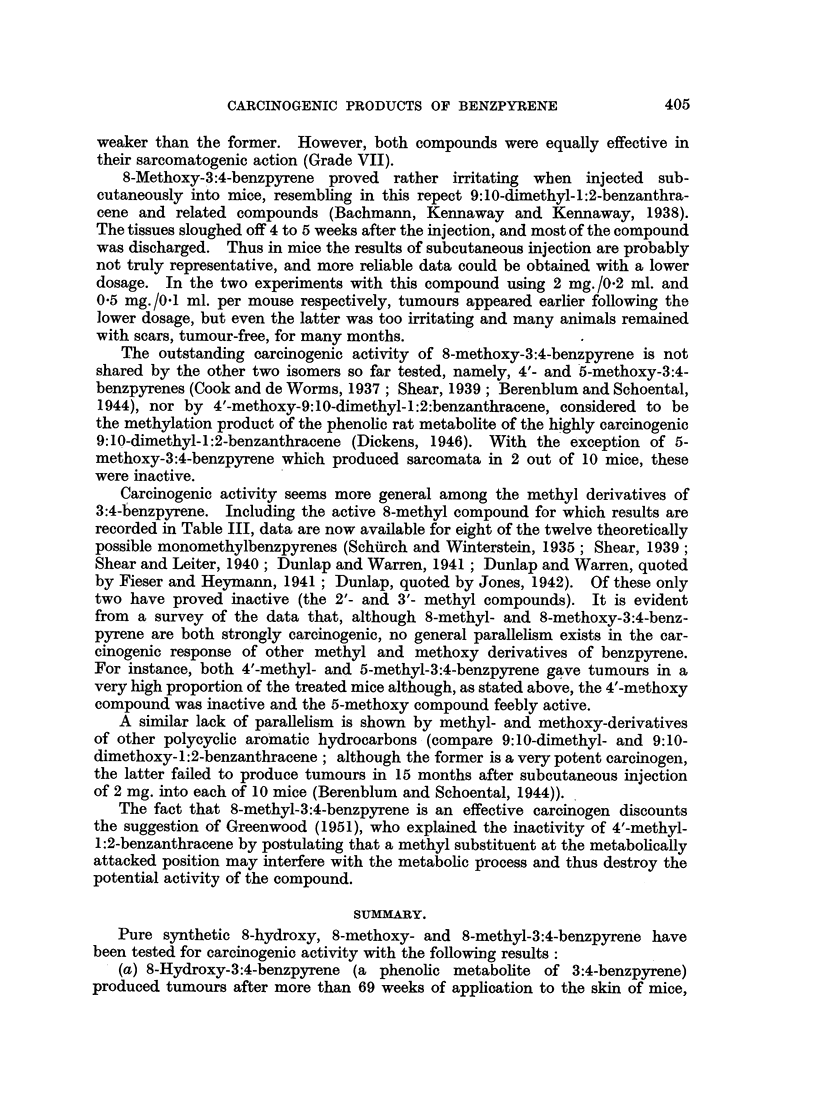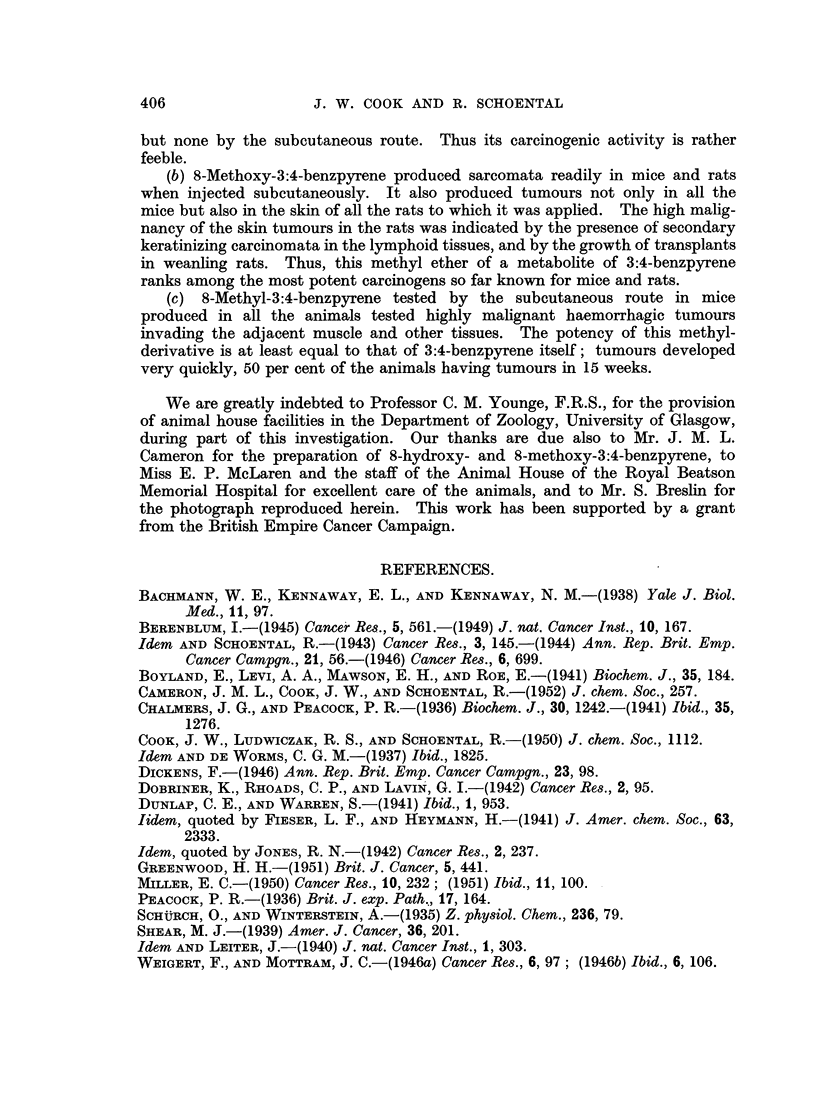# Carcinogenic Activity of Metabolic Products of 3:4-Benzpyrene: Application to Rats and Mice

**DOI:** 10.1038/bjc.1952.45

**Published:** 1952-12

**Authors:** J. W. Cook, R. Schoental

## Abstract

**Images:**


### CARCINOGENIC ACTIVITY OF METABOLIC PRODUCTS OF 3:4-BENZPYRENE: APPLICATION TO RATS AND MICE.

J. W. COOK AND R. SCHOENTAL.

*From the Chemistry Department, Glasgow University, and the Cancer Research Department, Royal Beatson Memorial Hospital, Glasgow.**

Received for publication October 24, 1952.

NEARLY a quarter of a century has elapsed since the first animal tumours were produced by the application of pure polycyclic aromatic hydrocarbons, and there is still complete ignorance as to the mechanism of local carcinogenic action. It is not known whether it is due to the intact hydrocarbon or to a metabolite, and which of the cell constituents are involved in the process.

Metabolic studies undertaken with a view to obtaining information as to the mechanism of the carcinogenic process have not yet solved the problem. They revealed, however, that the hydrocarbon undergoes metabolic changes, including oxidation to phenolic derivatives, quinones and acids formed by ring-degradation. Only a small fraction is eliminated in the form of unchanged hydrocarbon. The first step in the metabolic chain of events may be the binding of the hydrocarbon by a cell constituent (probably a protein) as suggested by the experiments of Weigert and Mottram (1946*a*, 1946*b*) and Miller (1950, 1951), followed by a local change to an acidic derivative, the nature of which is not yet known, as no pure products have so far been isolated from the metabolites occurring locally. Further changes seem to take place in the liver and during the passage through the intestines (Peacock, 1936 ; Chalmers and Peacock, 1936, 1941 ; Weigert and Mottram, 1946*a*, 1946*b*).

The metabolites of carcinogenic hydrocarbons, so far identified, have been isolated from the excreta of animals, but they are present in the circulating blood and may affect the carcinogenic process. It is, therefore, of importance to test the carcinogenic activity of each metabolite which becomes available in the pure state.

Only three phenolic metabolites of carcinogenic hydrocarbons have so far been tested for carcinogenic activity. The two dihydroxy-derivatives of 1:2:5:6-dibenzanthracene proved inactive (Boyland, Levi, Mawson and Roe, 1941 ; Dobriner, Rhoads and Lavin, 1942 ; Dunlap and Warren, 1941), while the crude fraction of phenolic metabolites of 3:4-benzpyrene gave one sarcoma in 10 mice tested by the subcutaneous route and one mammary tumour, the spontaneous origin of which could not be excluded (Berenblum and Schoental, 1943).

Since then, 8- and 10-hydroxy-derivatives have been identified as phenolic metabolites of 3:4-benzpyrene in mice, rats, and rabbits (Berenblum and Schoental, 1946), and their structures established by direct comparison of their respective methylation products with the synthetic 8- and 10-methoxy-3:4-benzpyrenes

* Formerly the Glasgow Royal Cancer Hospital.

(Cook, Ludwiczak and Schoental, 1950). The method of synthesis of 8-hydroxy-3:4-benzpyrene and its methyl ether enabled these pure compounds to be prepared in quantities adequate for biological testing. The results of these tests are now reported, as well as those with 8-methyl-3:4-benzpyrene, which has been synthesised for comparison with the 8-methoxy-compound (Cameron, Cook and Schoental, 1952).

### MATERIAL AND METHODS.

Mice were used for the testing of carcinogenic activity of the pure synthetic 8-hydroxy-, 8-methoxy- and 8-methyl-3:4-benzpyrene. 8-Methoxy-benzpyrene was tested also in rats.

The mice were derived from one pair of CBA strain and were bred locally, as were also the rats which were derived from a commercial stock of albino Wistar rats. The animals were housed in metal cages and received rat cake and tap water *ad libitum*.

Freshly prepared solutions (the solvents used were redistilled and freed from fluorescent impurities) of the pure crystalline substances (0·5 per cent solution in acetone in the case of 8-hydroxy-3-4-benzpyrene, a saturated solution in acetone or a 0·5 per cent solution in benzene in the case of 8-methoxy-3:4-benzpyrene) were applied twice a week to the intrascapular region of the skin from a capillary pipette, the hair being removed by clipping with scissors. For subcutaneous injections the pure compounds were dissolved in tricaprylin, and the animals were injected in the left flank, care being taken to prevent leakage of the oily solutions. In the majority of cases the injected oily solution formed a defined nodule palpable under the skin and remaining *in situ* for many weeks or months. The animals were allowed to live as long as possible ; but some were killed when moribund, or when the induced tumours attained excessive size. All the animals were examined *post mortem*, and the tumours and other observed abnormalities confirmed by histological examination. To obtain evidence of the degree of malignancy some of the tumours have been transplanted for several generations into the respective species of animal, and their histological appearance confirmed in the transplants.

### RESULTS.

Table I summarizes the results of testing of 8-hydroxy-3:4-benzpyrene. This compound did not produce any local tumours in the 16 mice (10 males and 6 females), into each of which it was injected subcutaneously in two doses, 2 mg. each at 5 months' interval, although 14 animals were alive after 52 weeks, 12 after 79 weeks, and the last 2 were killed when the experiment was terminated 117 weeks after the first injection. Several of the mice exhibited different degrees of leucoses which might have been spontaneous (in the control group of these CBA mice leucoses were also encountered), or accentuated by the action of this compound.

In the animals receiving skin applications of a 0·5 per cent solution of 8-hydroxy-3:4-benzpyrene in acetone (special care was taken to minimise the decomposition of this labile compound by dissolving only very small quantities at a time and keeping the solution in a dark bottle) the treated skin areas remained smooth and soft, and no abnormalities were visible during the first 69 weeks of treatment. However, on continued application small papillomata appeared, which grew progessively ; five of these became malignant. The experiment was terminated 121 weeks after the beginning of treatment ; about 50 per cent of the animals had some neoplastic local changes.

Table II gives the results of testing 8-methoxy-3:4-benzpyrene in mice and rats. This compound proved to be one of the strongest carcinogens so far known, probably exceeding in potency the parent 3:4-benzpyrene. Thus application of its saturated acetone solution (less than 0·2 per cent) to the skin of mice produced local depilation, dark pigmentation and desquamation a few days after the treatment began ; papillomata arose in multiple foci 10 weeks after the beginning of treatment, so that 50 per cent of the animals had tumours after 14 weeks, and 100 per cent after 22 weeks. Ten mice were killed after 27 weeks of treatment and 9 of these had multiple malignant tumours invading the underlying skeletal muscle ; one had only multiple papillomata. The experiment was terminated after 41 weeks, when the 4 remaining mice with carcinoma were killed. The papillomata from the multiple foçi gave in many cases big confluent malignant tumours occupying an area of up to 3 × 1·5 cm. at the site of application.

On subcutaneous injection of 2 mg. in 0·2 ml. of tricaprylin, 8-methoxy-3:4-benzpyrene proved very irritating and ulceration and discharge of the substance occurred in about 4 weeks after the injection ; this effect was more pronounced in the female series. Consequently tumours appeared later among the females (the first tumour appeared after 22 weeks, and 50 per cent of the animals had tumours after 26 weeks) than among the male mice (in which the first tumour appeared after 11 weeks and 50 per cent after 22 weeks). In another experiment 16 mice were injected each with 0·5 mg. of 8-methoxy-3:4-benzpyrene in 0·1 ml. of tricaprylin, but in spite of this reduced dosage some ulceration still occurred. In this series the first tumour appeared 10 weeks after the injection and 50 per cent of the animals had tumours in 14 weeks. However, in both series the extrusion of the material might have been responsible for the non-appearance of tumours in several animals. Still lower dosage would be necessary to obtain truly representative data as to the carcinogenic response of mice. In one case a tumour at the site of a scar appeared as late as 83 weeks after the injection.

No irritation was observed in the rats tested by the subcutaneous route. All the 11 animals developed sarcomata ; the first appeared in 16 weeks and 50 per cent of the rats had tumours 19 weeks after the first injection.

8-Methoxy-3:4-benzpyrene was tested also by skin application in rats. A saturated acetone solution applied twice weekly to the intrascapular region for 39 weeks did not seem to produce macroscopic changes. Subsequently a 0·5 per cent solution of this compound in benzene was applied. The treated area became depilated, scaly, and papillomata started to appear. It is not unlikely that the long induction period of these tumours (about 1 year) might have been shortened if the more concentrated benzene solution had been applied from the beginning of treatment. Once the papillomata appeared they readily became malignant, and keratinous horn-like structures grew to enormous size (5 × 5 × 4·5 cm.) between the ears of the rats. Several of these tumours metastasised to the local nymph nodes. The rat shown in Fig. 1 is a representative example. It shows besides the primary tumour a metastasis which formed a keratinised mass (1·5 × 1·5 cm.) in the submaxillary gland. Two of these tumours were transplanted into weanling rats, and carried successfully through three generations of transplants, which retained the ability to form keratin.

FIG. 1.—Horn-like tumour in a rat at the site of application of 8-methoxy-3:4-benzpyrene and metastasis in the submaxillary gland, 17 months after the first, and 3 months after the last application.

28

TABLE I.—*Incidence of Tumours in Mice Treated with 8-Hydroxy-3:4-benzpyrene.*

| Number and sex. | Dose. | Route. | Time of appearance of tumours. First. | Time of appearance of tumours. 50%. | Duration of experiment. | Number of tumours at site of application. | Other abnormalities. |
|---|---|---|---|---|---|---|---|
| 10 M. | 2 mg./0·2 ml. Second dose after 21 weeks | Subcutaneous | — | — | 91 weeks | 0 | 1 lymphosarcoma.<br>1 hyperplasia of lymph nodes. |
| 6 F. | Ditto | „ | — | — | 117 „ | 0 | 3 leucoses.<br>1 lung adenoma. |
| 10 M. | 0·5% in acetone twice weekly | Skin | 69 weeks | 113 weeks | 117 „ | 3 carcinomas<br>3 papillomas | 4 leucoses.<br>3 hepatomas.<br>1 adenocarcinoma of lung. |
| 10 F. | Ditto | „ | 69 „ | 113 „ | 121 „ | 2 carcinomas<br>3 papillomas | 3 leucoses.<br>2 lung adenomas. |

TABLE II.—*Incidence of Tumours in Mice and Rats Treated with 8-Methoxy-3:4-benzpyrene.*

| Animal. | Number and sex. | Dose. | Route. | Time of appearance of tumours. First. | Time of appearance of tumours. 50%. | Duration of experiment. | Number of tumours at site of application. | Other abnormalities. |
|---|---|---|---|---|---|---|---|---|
| Rats | 11 M. | 2 mg./0·2 ml. Second dose after 13 weeks | Subcutaneous | 16 weeks | 19 weeks | 30 weeks | 11 sarcomas | — |
| „ | 5 F. | Saturated solution in acetone for 39 weeks, then 0·5% in benzene twice weekly | Skin | 44 „ | 50 „ | 87 „ | 5 carcinomas | 2 metastases in lymph nodes. |
| „ | 3 M. | | „ | 48 „ | 48 „ | 87 „ | 3 „ | 1 metastasis in submaxillary gland.<br>1 metastasis in parotid gland. |
| Mice | 9 M. | 2 mg./0·2 ml. | Subcutaneous | 11 „ | 22 „ | 67 „ | 9 sarcomas | — |
| „ | 12 F. | „ „ | „ | 22 „ | 26 „ | 65 „ | 7 „ | — |
| „ | 16 F. +M. | 0·5 mg./0·1 ml. | „ | 10 „ | 14 „ | 95 „ | 4 „<br>4 „ and carcinomas | 5 leucoses.<br>2 lung adenomas.<br>1 hepatoma.<br>1 metastasis in peritoneum and pelvic organs.<br>2 bronchopneumonias |
| „ | 21 F. | Saturated solution in acetone twice weekly | Skin | 10 „ | 14 „ | 41 „ | 20 carcinomas<br>1 papilloma | 2 leucoses. |

Table III gives the results of subcutaneous testing of 8-methyl-3:4-benzpyrene in mice. At the dosage of 1 mg. in 0·1 ml. tricaprylin this compound produced

TABLE III.—*Incidence of Tumours in Mice Injected Subcutaneously with 8-Methyl-3:4-benzpyrene.*

| Number and sex. | Dose. | Time of appearance of tumours. First. | 50%. | Duration of experiment. | Number of tumours. |
|---|---|---|---|---|---|
| 11 M. | 1 mg./0·1 ml. | 13 weeks | 14 weeks | 27 weeks | 11 sarcomas. |
| 7 F. | ditto | 13 „ | 15 „ | 27 „ | 7 „ |

some local irritation ; the first tumour appeared after 13 weeks, and 50 per cent of the mice had tumours by about 14 weeks after the injections. All the 18 injected mice developed highly malignant tumours invading the adjacent muscle and other tissues. One of these tumours was carried through 3 generations of transplants, retaining its original structure.

### DISCUSSION.

The results here reported present several points of interest.

(*a*) It is the first time that carcinogenic activity has been demonstrated by the application to the skin of a phenolic metabolite of a carcinogenic hydrocarbon (pure 8-hydroxy-3:4-benzpyrene) in mice. However, the very long induction period (69 weeks for the appearance of the first papilloma and 113 weeks for tumours to develop in 50 per cent of the animals) corresponds with or rather exceeds the survival times of mice in the carcinogenic tests with the other known metabolites. Hence a low degree of carcinogenic activity such as is exhibited by 8-hydroxy-3:4-benzpyrene may not be as isolated a phenomenon as it seems at present.

No tumours were produced by subcutaneous injection of this compound, which in conjunction with the above results makes it obvious that the carcinogenicity of the parent hydrocarbon is considerably reduced (although not completely abolished) by its metabolic transformation into a phenolic derivative. Of the other benzpyrenols so far tested the 4′- (Cook and de Worms, 1937 ; Shear, 1939), 2- (Dunlap and Warren, 1941) and 5- (Shear and Leiter, 1940) compounds were inactive, while the 6-isomer produced tumours in 2 out of 40 mice when injected subcutaneously (Dunlap and Warren, 1941).

(*b*) Of particular interest is the finding that 8-methoxy-3:4-benzpyrene exhibits a sarcomatogenic and carcinogenic activity of the highest order in both the species tested, mice and rats. While rats are known to develop sarcomata readily on subcutaneous injections of carcinogens, not many skin tumours have been produced in rats by the application of tar or pure carcinogenic hydrocarbons. Berenblum (1949) summarized the relevant literature when he reported on the carcinogenicity of 9:10-dimethyl-1:2-benzanthracene. The latter proved very effective for rat skin, giving rise to 14 tumours (11 of these were malignant) in 20 rats when applied once a week as a 0·5 per cent solution in benzene during 45 weeks.

By comparison with 9:10-dimethyl-1:2-benzanthracene (potency Grade VI, when graded according to the system of Berenblum (1945)), 8-methoxy-3:4-benzpyrene showed towards the skin of rats the potency Grade V, only a little weaker than the former. However, both compounds were equally effective in their sarcomatogenic action (Grade VII).

8-Methoxy-3:4-benzpyrene proved rather irritating when injected subcutaneously into mice, resembling in this repect 9:10-dimethyl-1:2-benzanthracene and related compounds (Bachmann, Kennaway and Kennaway, 1938). The tissues sloughed off 4 to 5 weeks after the injection, and most of the compound was discharged. Thus in mice the results of subcutaneous injection are probably not truly representative, and more reliable data could be obtained with a lower dosage. In the two experiments with this compound using 2 mg./0·2 ml. and 0·5 mg./0·1 ml. per mouse respectively, tumours appeared earlier following the lower dosage, but even the latter was too irritating and many animals remained with scars, tumour-free, for many months.

The outstanding carcinogenic activity of 8-methoxy-3:4-benzpyrene is not shared by the other two isomers so far tested, namely, 4′- and 5-methoxy-3:4-benzpyrenes (Cook and de Worms, 1937 ; Shear, 1939 ; Berenblum and Schoental, 1944), nor by 4′-methoxy-9:10-dimethyl-1:2:benzanthracene, considered to be the methylation product of the phenolic rat metabolite of the highly carcinogenic 9:10-dimethyl-1:2-benzanthracene (Dickens, 1946). With the exception of 5-methoxy-3:4-benzpyrene which produced sarcomata in 2 out of 10 mice, these were inactive.

Carcinogenic activity seems more general among the methyl derivatives of 3:4-benzpyrene. Including the active 8-methyl compound for which results are recorded in Table III, data are now available for eight of the twelve theoretically possible monomethylbenzpyrenes (Schürch and Winterstein, 1935 ; Shear, 1939 ; Shear and Leiter, 1940 ; Dunlap and Warren, 1941 ; Dunlap and Warren, quoted by Fieser and Heymann, 1941 ; Dunlap, quoted by Jones, 1942). Of these only two have proved inactive (the 2′- and 3′- methyl compounds). It is evident from a survey of the data that, although 8-methyl- and 8-methoxy-3:4-benzpyrene are both strongly carcinogenic, no general parallelism exists in the carcinogenic response of other methyl and methoxy derivatives of benzpyrene. For instance, both 4′-methyl- and 5-methyl-3:4-benzpyrene gave tumours in a very high proportion of the treated mice although, as stated above, the 4′-methoxy compound was inactive and the 5-methoxy compound feebly active.

A similar lack of parallelism is shown by methyl- and methoxy-derivatives of other polycyclic aromatic hydrocarbons (compare 9:10-dimethyl- and 9:10-dimethoxy-1:2-benzanthracene ; although the former is a very potent carcinogen, the latter failed to produce tumours in 15 months after subcutaneous injection of 2 mg. into each of 10 mice (Berenblum and Schoental, 1944)).

The fact that 8-methyl-3:4-benzpyrene is an effective carcinogen discounts the suggestion of Greenwood (1951), who explained the inactivity of 4′-methyl-1:2-benzanthracene by postulating that a methyl substituent at the metabolically attacked position may interfere with the metabolic process and thus destroy the potential activity of the compound.

### SUMMARY.

Pure synthetic 8-hydroxy, 8-methoxy- and 8-methyl-3:4-benzpyrene have been tested for carcinogenic activity with the following results :

(*a*) 8-Hydroxy-3:4-benzpyrene (a phenolic metabolite of 3:4-benzpyrene) produced tumours after more than 69 weeks of application to the skin of mice, but none by the subcutaneous route. Thus its carcinogenic activity is rather feeble.

(*b*) 8-Methoxy-3:4-benzpyrene produced sarcomata readily in mice and rats when injected subcutaneously. It also produced tumours not only in all the mice but also in the skin of all the rats to which it was applied. The high malignancy of the skin tumours in the rats was indicated by the presence of secondary keratinizing carcinomata in the lymphoid tissues, and by the growth of transplants in weanling rats. Thus, this methyl ether of a metabolite of 3:4-benzpyrene ranks among the most potent carcinogens so far known for mice and rats.

(*c*) 8-Methyl-3:4-benzpyrene tested by the subcutaneous route in mice produced in all the animals tested highly malignant haemorrhagic tumours invading the adjacent muscle and other tissues. The potency of this methyl-derivative is at least equal to that of 3:4-benzpyrene itself; tumours developed very quickly, 50 per cent of the animals having tumours in 15 weeks.

We are greatly indebted to Professor C. M. Younge, F.R.S., for the provision of animal house facilities in the Department of Zoology, University of Glasgow, during part of this investigation. Our thanks are due also to Mr. J. M. L. Cameron for the preparation of 8-hydroxy- and 8-methoxy-3:4-benzpyrene, to Miss E. P. McLaren and the staff of the Animal House of the Royal Beatson Memorial Hospital for excellent care of the animals, and to Mr. S. Breslin for the photograph reproduced herein. This work has been supported by a grant from the British Empire Cancer Campaign.

### REFERENCES.

Bachmann, W. E., Kennaway, E. L., and Kennaway, N. M.—(1938) *Yale J. Biol. Med.*, **11**, 97.

Berenblum, I.—(1945) *Cancer Res.*, **5**, 561.—(1949) *J. nat. Cancer Inst.*, **10**, 167.

*Idem* and Schoental, R.—(1943) *Cancer Res.*, **3**, 145.—(1944) *Ann. Rep. Brit. Emp. Cancer Campgn.*, **21**, 56.—(1946) *Cancer Res.*, **6**, 699.

Boyland, E., Levi, A. A., Mawson, E. H., and Roe, E.—(1941) *Biochem. J.*, **35**, 184.

Cameron, J. M. L., Cook, J. W., and Schoental, R.—(1952) *J. chem. Soc.*, 257.

Chalmers, J. G., and Peacock, P. R.—(1936) *Biochem. J.*, **30**, 1242.—(1941) *Ibid.*, **35**, 1276.

Cook, J. W., Ludwiczak, R. S., and Schoental, R.—(1950) *J. chem. Soc.*, 1112.

*Idem* and de Worms, C. G. M.—(1937) *Ibid.*, 1825.

Dickens, F.—(1946) *Ann. Rep. Brit. Emp. Cancer Campgn.*, **23**, 98.

Dobriner, K., Rhoads, C. P., and Lavin, G. I.—(1942) *Cancer Res.*, **2**, 95.

Dunlap, C. E., and Warren, S.—(1941) *Ibid.*, **1**, 953.

*Iidem*, quoted by Fieser, L. F., and Heymann, H.—(1941) *J. Amer. chem. Soc.*, **63**, 2333.

*Idem*, quoted by Jones, R. N.—(1942) *Cancer Res.*, **2**, 237.

Greenwood, H. H.—(1951) *Brit. J. Cancer*, **5**, 441.

Miller, E. C.—(1950) *Cancer Res.*, **10**, 232 ; (1951) *Ibid.*, **11**, 100.

Peacock, P. R.—(1936) *Brit. J. exp. Path.*, **17**, 164.

Schürch, O., and Winterstein, A.—(1935) *Z. physiol. Chem.*, **236**, 79.

Shear, M. J.—(1939) *Amer. J. Cancer*, **36**, 201.

*Idem* and Leiter, J.—(1940) *J. nat. Cancer Inst.*, **1**, 303.

Weigert, F., and Mottram, J. C.—(1946*a*) *Cancer Res.*, **6**, 97 ; (1946*b*) *Ibid.*, **6**, 106.